# Enhanced food-related responses in the ventral medial prefrontal cortex in narcolepsy type 1

**DOI:** 10.1038/s41598-018-34647-6

**Published:** 2018-11-06

**Authors:** Ruth Janke van Holst, Lieneke K. Janssen, Petra van Mierlo, Gert Jan Lammers, Roshan Cools, Sebastiaan Overeem, Esther Aarts

**Affiliations:** 1 Department of Neurology, Radboud university medical center, Nijmegen, The Netherlands; 2 Donders Institute for Brain, Cognition and Behaviour, Radboud University, Nijmegen, The Netherlands; 3 Sleep Medicine Center Kempenhaeghe, Heeze, The Netherlands; 4 Sleep-Wake Center SEIN, Heemstede, The Netherlands; 5 Department of Neurology, Leiden University Medical Center, Leiden, The Netherlands; 6 Department of Psychiatry, Radboud university medical center, Nijmegen, The Netherlands; 7 Eindhoven University of Technology, Eindhoven, The Netherlands; 8 Department of Psychiatry, Amsterdam UMC, University of Amsterdam, Amsterdam, The Netherlands

**Keywords:** Neurological disorders, Sleep disorders

## Abstract

Narcolepsy type 1 is a chronic sleep disorder caused by a deficiency of the orexin (hypocretin) neuropeptides. In addition to sleep regulation, orexin is important for motivated control processes. Weight gain and obesity are common in narcolepsy. However, the neurocognitive processes associated with food-related control and overeating in narcolepsy are unknown. We explored the neural correlates of general and food-related attentional control in narcolepsy-type-1 patients (n = 23) and healthy BMI-matched controls (n = 20). We measured attentional bias to food words with a Food Stroop task and general executive control with a Classic Stroop task during fMRI. Moreover, using multiple linear regression, we assessed the relative contribution of neural responses during Food Stroop and Classic Stroop to spontaneous snack intake. Relative to healthy controls, narcolepsy patients showed enhanced ventral medial prefrontal cortex responses and connectivity with motor cortex during the Food Stroop task, but attenuated dorsal medial prefrontal cortex responses during the Classic Stroop task. Moreover, the former activity but not the latter, was a significant predictor of spontaneous snack intake. These findings demonstrate that narcolepsy, characterized by orexin deficiency, is associated with decreased dorsal medial prefrontal cortex responses during general executive control and enhanced ventral medial prefrontal cortex responses during food-driven attention.

## Introduction

Narcolepsy type 1 (NT1) is a disabling sleep disorder, primarily characterized by excessive daytime sleepiness and emotionally triggered episodes of muscle weakness called cataplexy. The disorder is caused by a loss of orexin (hypocretin)–producing neurons located in the lateral hypothalamus. Orexin neurotransmission mediates behavior under situations of high motivational relevance, through excitatory influences on the histaminergic, monoaminergic, and cholinergic system^1^. Interestingly, the incidence of obesity is twice as high in narcolepsy compared with the normal population^2–7^. We recently showed that food-specific satiety had reduced effects on food choices and caloric intake in NT1 patients, suggesting an important functional role for orexin in human food-related control of behaviour^8^. However, the neurocognitive processes associated with food-related control and overeating in orexin-deficient patients are unknown.

Enhanced attention towards food over non-food information (i.e. attentional bias) has been proposed to contribute to the development and/or maintenance of obesity (e.g. for a review see^9^). Functional MRI studies revealed that food cues relative to neutral cues can elicit enhanced activation of the reward regions in the mesolimbic dopamine pathway in overweight relative to healthy weight individuals^10–12^, including the ventral medial prefrontal cortex (vmPFC), striatum, insula and amygdala, which might drive excessive attention towards food cues. Detecting food rapidly and maintaining attention on food could increase the likelihood of overeating and, in the long term, obesity^13–15^. In addition, loss of executive control during food-related distraction has been related to obesity^16^. Although obesity is a common symptom in narcolepsy and orexin neurons interact with the mesolimbic dopamine system^17–19^, it is unclear whether narcolepsy patients show abnormal attentional bias toward food cues, and what neurocognitive mechanism would underlie this effect. We therefore used a Food Stroop task (i.e. measuring reaction times toward food-related words and neutral words)^16,20^ during fMRI in NT1 patients compared with healthy BMI-matched controls. We previously showed that spontaneous snack intake was increased in narcolepsy versus controls in a largely overlapping sample^8^, and therefore investigated whether brain responses on the Food Stroop would relate to this snack intake. Additionally, we applied a Classic Stroop task (i.e. measuring response conflict) to assess general executive control abilities and evaluated the relative contribution of the neural findings on the Classic Stroop and Food Stroop tasks to spontaneous snack intake.

## Methods and Materials

### Participants

Forty-three right-handed participants were included in the experiment (20 healthy controls, 23 NT1 patients). Patients were recruited from the Dutch national expertise centers for narcolepsy at Sleep Medicine Center Kempenhaeghe (Heeze, the Netherlands) and Sleep-wake Center SEIN (Heemstede, the Netherlands); as well as through advertisement by the Dutch narcolepsy patients’ organization. Healthy control participants were recruited via poster and word-of-mouth advertisements in Nijmegen and surrounding areas. Healthy controls were matched to the NT1 patients in terms of average age, gender, BMI and level of education.

Inclusion criteria were age 18–60 years old, BMI 20–35 and right-handedness. Exclusion criteria were diabetes mellitus, (a history of) clinically significant hepatic, cardiac, renal, cerebrovascular, endocrine, metabolic or pulmonary disease, uncontrolled hypertension, (a history of) clinically significant neurological or psychiatric disorders and current psychological treatment other than for narcolepsy or idiopathic hypersomnia, deafness, blindness, or sensory-motor handicaps, history of taste or smell impairments, drug, alcohol or gamble addiction in the past 6 months, inadequate command of Dutch language, current presence of an eating disorder, current strict dieting (i.e. calorie-restricted diet and/or in treatment with dietician), or food allergy to one of the ingredients used in the experiment.

All patients were diagnosed according to the International Classification of Sleep Disorders – Third Edition (ICSD-3), by a narcolepsy expert (SO or GJL). All fulfilled the criteria for NT1, including the presence of clear-cut cataplexy as well as a low mean sleep latency (<8 minutes) measured with the Multiple Sleep Latency Test (MSLT) and at least 2 sleep onset REM periods (SOREMPs) during MLST naps and the previous night’s diagnostic sleep study. Other sleep disorders including sleep deprivation were ruled out. In 13 patients, orexin cerebrospinal fluid levels were known and shown to be equal or lower than 110 pg/ml.

We also compared NT1 patients with an additional control group, namely patients with idiopathic hypersomnia (IH, n = 15), to verify that our findings in narcolepsy patients were not solely attributable to possible decreased alertness and/or medication-withdrawal. Patients with idiopathic hypersomnia all had clear excessive daytime sleepiness, a mean sleep latency at the MSLT of 8 minutes or less, and the symptoms were not explained by another sleep disorder. Because of the rareness of the disorder, this small convenience sample could not be perfectly matched with the other two experimental groups (see Supplementary Information Table 1). All participants were recruited on a voluntary basis and gave written informed consent before the start of the study. The study was approved by the Ethical Committee of the Radboud university medical center (CMO Arnhem-Nijmegen) and reported in the acknowledged Dutch Trial register (www.trialregister.nl: TC = 4508) and all experiments were performed in accordance with relevant guidelines and regulations.

### Food Stroop task and Classic Stroop task

**Figure 1:**
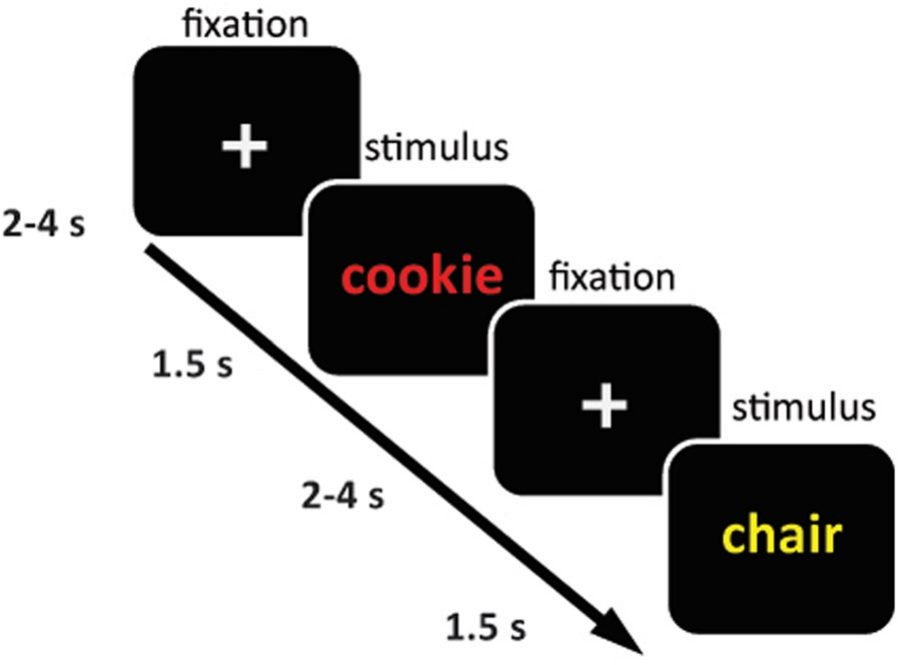
Sample trial of the Food Stroop task. On each trial, participants indicated the color of the word presented on the screen by pressing the button reflecting that color. Participants were presented with food and neutral words.

Subjects were instructed in both tasks before going into the scanner and were further familiarized with the task by practicing the color-button contingency and performing 10 practice trials with feedback (correct/incorrect) in the scanner. For task details see Fig. 1. In short, subjects had to indicate the color of the word presented on the screen pressing the button reflecting that color as fast and accurately as possible. In the Food Stroop task, subjects were presented with food words and neutral words, whereas in the Classic Stroop task, subjects were presented with congruent color words (e.g. the word “GREEN” printed in green) or incongruent color words (e.g. the word “GREEN” printed in red). The tasks were programmed in Presentation software (Neurobehavioral Systems Inc. htpps://www.neurobs.com). All task stimuli were presented with a digital projector on a screen at the back end of the MRI scanner bore, which was visible via a mirror mounted on the head coil. Responses were made using an MRI-compatible button box. Twenty generally high-calorie, palatable food words were selected from word lists reported in previous studies^20,21^. Food words were matched to twenty neutral words each in terms of word length, number of syllables and frequency of use according to the SUBTLEX-NL norms^22^.

The Food Stroop interference score was calculated by subtracting the response time (RT) to neutral words from the RT to food words. Hence a higher interference score indicates more distraction by food words. Similarly, the Classic Stroop interference score was calculated by subtracting the response time (RT) to congruent words from the RT to incongruent words. Thus a higher Classic Stroop interference score indicates less general executive control ability.

### Ab-libitum snack intake

After the fMRI session, participants were asked to fill out questionnaires whilst four bowls with a variety of snacks were placed in front of them (see^8^ for the results in a largely overlapping, but larger sample). The four bowls contained: crisps, raisins, wine gums and cocktail nuts. They were told that they could eat the snacks if they felt like it. Unbeknownst to participants, we calculated the amount of kilocalories (kcal) consumed by weighting the bowl before and after, and by multiplying the amount of grams consumed by the amount of kcal/gram of that particular snack.

### Study procedure

Patients were asked to refrain from using their medication, if any, one week prior to the test day. On the day preceding the test day, all participants had to refrain from alcohol and drug intake, and participants had to refrain from smoking on the test day itself. Furthermore, participants fasted for at least 5 hours before the test session to ensure that they were motivated by food and snacks. The test session took place between 9am and 6 pm, and we aimed to test all participants in the morning, although due to practical reasons this was not feasible in all subjects. Timing of the session was matched between groups, with the majority of participants tested in the morning (starting at 9:00 till 13:00). There were, however, a few exceptions of participants tested in the afternoon (HC = 5, NT1 = 7, IH = 4). During the test day (3.5 hours in total) the participants completed questionnaires (e.g. Epworth Sleepiness Scale^23^ and Pittsburgh Sleep Quality Index^24^ and the digit span to assess working memory capacity and the the Dutch Eating Behavior Questionnaire (DEBQ)^25^ to assess emotional, external and restraint eating behaviour, and performed the Food Stroop task, directly followed by the classic color-word Stroop task during the MRI session. The test day was concluded by a behavioral satiation task and questionnaires while participants had access to ad libitum snacks; results from these measures were reported in a previous study^8^. The number of participants included in the current analyses is smaller and not completely overlapping with the previous study because some people who did complete the satiation task did not have usable scan data (NT1 n = 1) and vice versa (HC n = 1, NT1 n = 1).

### Behavioral Data Analysis

The mean latencies of the correct responses to the words and the number of correct responses in the tasks were analyzed with SPSS. We excluded trials with a RT < 200 msec.

Two narcolepsy patients (scoring 0% and 5% accuracy) scored <=10% on accuracy on the incongruent trials in the Classic Stroop task, resulting in too small number of trials to include in the fMRI analyses. These patients were therefore excluded from the Classic Stroop analyses (remaining NT1 group of n = 21), though they were included in the Food Stroop analyses. Behavioral group analyses including these outliers indicated no qualitatively different results on the Classic Stroop task compared with excluding these outliers (data not shown).

Two IH patients (scoring both 10% accuracy) were excluded from the Classic Stroop analyses (remaining NT1 group of n = 21 and IH group n = 13), though they were included in the Food Stroop analyses.

Response times were reciprocal-transformed (the reciprocal, x to 1/x, is a strong transformation which can be used on highly skewed data^26^ to assure that all assumptions of parametric data are met). All behavioral outcome measures were tested for and met the homogeneity of variance assumption. Repeated measurement ANOVAs were used for the two Stroop tasks separately, to test the main effect of Condition (Food Stroop: food, neutral; Classic Stroop: incongruent, congruent), Group (NT1, healthy controls), and Group * Condition interaction effects.

### Functional Imaging

Whole-brain imaging was performed on a 3 Tesla Siemens MR scanner located at the Donders Centre for Cognitive Neuroimaging, Nijmegen, The Netherlands. BOLD-sensitive functional images were acquired using a gradient-echo planar multi-echo scanning sequence (TR: 2070 ms; TEs for 4 echoes: 9 ms, 19.25 ms, 29.5 ms and 39.75 ms). We used a multi-echo EPI sequence to reduce image distortion and increase BOLD sensitivity in regions which are typically affected by strong susceptibility artifacts, such as the ventral striatum and vmPFC^27^. One volume consisted of 34 axial slices (voxel size: 3.5 × 3.5 × 3.0 mm^3^, field of view: 224 mm, flip angle: 90°). After acquisition of the functional images, a high-resolution anatomical scan (T1-weighted MP-RAGE, TR: 2300 ms, TE: 3.03 ms, 8° flip-angle, 192 sagittal slices, slice-matrix size: 256 × 256, voxel size: 1 × 1 × 1 mm^3^) was obtained. Total duration of MRI sessions was 45–60 minutes.

Data were pre-processed and analyzed using SPM8 (www.fil.ion.ucl.ac.uk/spm). The volumes for each echo time were realigned to correct for motion (estimation of the realignment parameters was done for the first echo and then copied to the other echoes). The four echo images were combined into a single MR volume based on 31 volumes acquired before the actual experiment started using an optimised echo weighting method. Combined functional images were slice-time corrected by realigning the time-series for each voxel temporally to acquisition of the middle slice. Structural and functional data were then co-registered and spatially normalised to a standardized stereotactic space (Montreal Neurological Institute (MNI) template). After segmentation of the structural images using a unified segmentation approach, the mean of the functional images was spatially coregistered to the bias-corrected structural images. The transformation matrix resulting from segmentation was then used to normalize the final functional images into MNI space (resampled at voxel size 2 × 2 × 2 mm). Finally, the normalised functional images were spatially smoothed using an isotropic 8 mm full-width at half-maximum Gaussian kernel.

### Functional MRI Data Analysis

Statistical analyses were performed according a general linear model (GLM) as implemented in SPM8. At the first level, subject-specific data were analyzed using a fixed effects model which contained 2 regressors of interest with the correct trials on food trials and those on neutral trials of the Food Stroop task and 2 regressors with the correct trials on incongruent trials and those on congruent trials of the Classic Stroop task. All onsets were modeled using a stick function and convolved with the canonical hemodynamic response function. We also included regressors of non-interest: one for incorrect trials, one for missed trials, as well as six movement parameters - resulting from the realignment procedure - and their six time derivatives to account for head movement, and finally the average ‘out of brain’ signal, derived from the segmented anatomical scan. High pass filtering (128 seconds) was applied to the time series of the functional images to remove low-frequency drifts and correction for serial correlations was done using an autoregressive AR(1) model.

At the second level, we investigated whole-brain main effect of the tasks and group effects in a random effects analysis. Group differences in brain responses on the Food Stroop (food – neutral) and on the Classic Stroop (incongruent – congruent) contrast were tested with an independent two-sample t-test; the main effects of the tasks were tested with a one-sample t-test. In all second level analyses, we added as covariate of non-interest a summary motion score for every subject, which was calculated as the sum of the root-mean-square value of the subject’s frame wise-displacement parameters (x, y, z in mm & pitch, roll, and yaw in degrees)^28^. We tested for correlations between whole-brain responses to food vs neutral words and spontaneous snack intake, as well as BMI, across healthy controls and NT1 patients. Additionally, we assessed the relative contribution of the neural findings (by extracting beta’s from the relevant clusters) on the Food Stroop and Classic Stroop tasks to spontaneous snack intake across healthy control and NT1 groups by using them as predictors in a multiple regression model in SPSS using the forced entry (or Enter as it is known in SPSS and using a p < 0.05 to report significant results) method. For the fMRI analyses we used an FWE-corrected cluster level threshold p < 0.05 (intensity threshold, uncorrected p < 0.001).

### Generalized Psycho-Physiological Interaction (gPPI) Analysis

**Figure 2:**
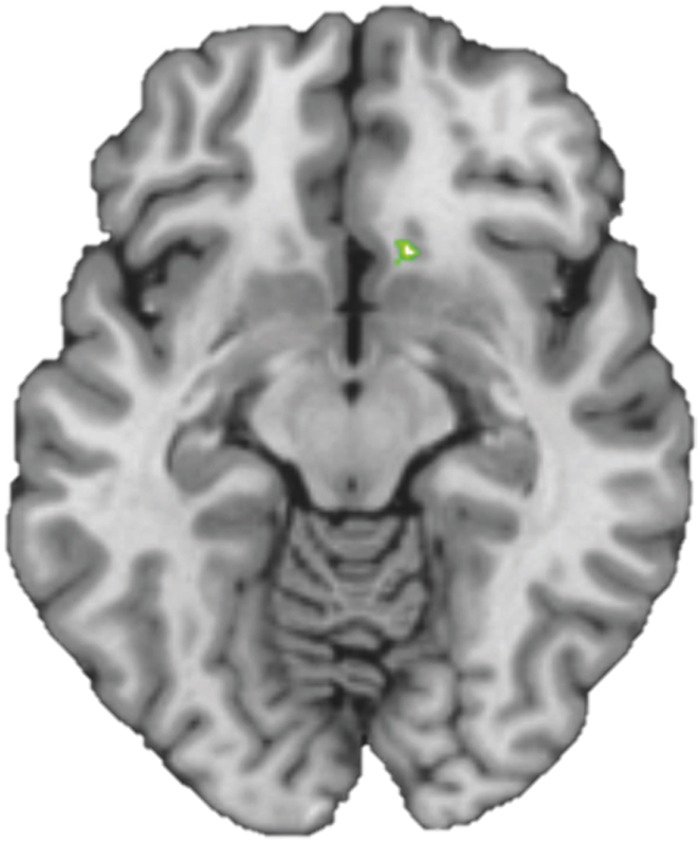
The right vmPFC seed, defined as the significant cluster from the food – neutral trials contrast indicating more activity in Narcolepsy type 1 patients relative to healthy controls (Fig. 4B), combined with the corresponding Automated Anatomical Labeling (AAL) masks.

To test functional connectivity differences between groups during color-naming of food versus neutral words, we conducted a generalized psychophysiological interaction analysis^29^. As a seed for the gPPI analyses we used the one cluster that was significantly different between the healthy controls and narcolepsy patients (see Results) during the Food Stroop task (i.e. the right ventral medial prefrontal cortex). See Fig. 2 for details and the resulting seed. Because we modeled the main effect of task in the PPI analysis, the PPI will only detect functional connectivity effects over and above (orthogonal to) the main effect of task, thus there is no concern about non-independence or circularity in this case^30^.

We used the generalized PPI toolbox (gPPI; http://www.nitrc.org/projects/gppi; McLaren *et al*.^29^) in SPM8 (Statistical Parametric Mapping, Wellcome Department of Cognitive Neurology, London, UK), given that gPPI has the flexibility to accommodate multiple task conditions in the same connectivity model. To estimate the neural activity producing the physiological effect in the seed region for each subject, the BOLD signal was extracted from this region and deconvolved^31^. This was included in the model as a physiological regressor, as were the onset times for each of the task conditions (food and neutral) as psychological regressors, as well as the physiological regressor multiplied by the psychological regressors (convolved with the HRF), resulting in 5 regressors on the first level (i.e., one physiological, 2 psychological, and 2 interaction regressors). One PPI contrast was created for each subject: food trials – neutral trials. On the second level, this PPI contrast was analyzed using independent two-sample t-tests comparing healthy controls with NT1 patients. We used an FWE-corrected cluster level threshold of p < 0.05 (intensity threshold uncorrected p < 0.001).

## Results

### Participants

Table 1 summarizes the demographic and clinical characteristics of the participants who were included in the data analysis. Narcolepsy patients and healthy controls were well matched on gender, age, BMI and education level and both scored average/normal relative to norm scores on restraint, emotional, and external eating as measured with the DEBQ^25^.

**Table 1:** Demographic and clinical characteristics.

	Controls (n = 20)	NT1 patients (n = 23)	STATS
Male/Female	10/10	12/11	p = 0.887
Age	36.75 (12.14)	33.83 (8.36)	p = 0.358
Total score Digit Span	16.20 (4.10)	15.13 (3.36)	p = 0.353
Education levels:	2.95 (0.95)	2.78 (0.80)	p = 0.538
BMI	25.30 (3.84)	26.70 (3.95)	p = 0.245
Disease duration	—	8.17 (8.29)	—
ESS	6.40 (3.66)	16.13 (4.84)	p < 0.001*
PSQI	4.75 (3.85)	7.39 (2.31)	P = 0.009*
DEBQ restrained	2.97 (1.01)	2.76 (0.70)	P = 0.899
DEBQ emotional	2.49 (1.01)	2.77 (0.87)	P = 0.346
DEBQ external	2.88 (0.45)	3.05 (0.48)	P = 0.224
Medication used			
Stimulants^#^	—	13	—
Anti-depressants^#^	—	1	
Sodium oxybate^#^	—	3	
Stimulants plus sodium oxybate^#^	—	2	
No medication^#^	20	4	

Note. Variables are reported as mean and (standard deviations). Disease duration is reported in mean years. Education levels were categorized as 1 = Lower Vocational Education, 2 = Intermediate Vocational, 3 = Higher Vocational, 4 = University; BMI = Body Mass Index; ESS = Epworth Sleepiness Scale; PSQI = Pittsburgh Sleep Quality Index; ^#^number of participants; *Significant at < 0.05; Group differences on age, gender, education level and medication use were tested with an Chi-square test. Other tests were F-tests. NT1: narcolepsy type 1 patients.

### Ad libitum food intake

Narcolepsy patients spontaneously consumed significantly more calories (mean: 324.71 SD: 272.20) during the ad-libitum snack intake than healthy controls (mean: 114.29 SD: 150.46; F(1,42) = 11.108 p = 0.002).

### Behavioral performance on the Food Stroop task

**Figure 3:**
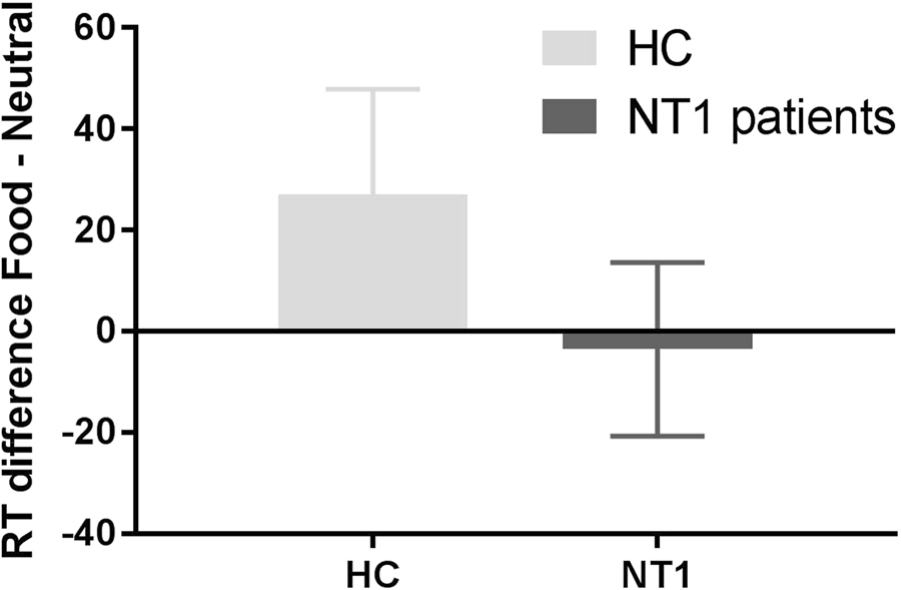
Reaction times during the Food Stroop task. Values are RT means for the difference between food and neutral words. Bars indicate standard errors of the group mean. HC = healthy controls; NT1 patients = Narcolepsy type 1 patients. RT = reaction time; ms = milliseconds.

Participants did not respond faster to food words than to neutral words (main effect of Condition: F(1,39) = 1.767, p = 0.192). We did not see a main effect of Group: (F(1,39) = 0.941, p = 0.338), and no significant Condition * Group effect on RTs (F(1,39) = 1.354, p = 0.257) (Table 2; Fig. 3).

**Table 2:** Behavioral results from the Food Stroop task and classic Stroop task.

Food Stroop task	Food RTs (ms)	Neutral RTs (ms)	Food Stroop RT effect (ms) (food – neutral)	Food accuracy (%)	Neutral accuracy (%)	Food Stroop accuracy effect (%) (neutral –food)
Healthy controls (n = 20)	976.73 (376.85)	949.62 (374.34)	27.11 (92.91)	97.88 (2.37)	97.12 (4.00)	0.75 (4.45)
NT1 patients (n = 23)	811.30 (109.15)	814.86 (116.30)	−3.56 (85.45)	96.85 (3.30)	97.50 (2.72)	−0.65 (4.54)
**Classic Stroop Task**	**Congruent RTs (ms)**	**Incongruent RTs (ms)**	**Stroop RT effect (ms) (incongruent – congruent)**	**Congruent accuracy (%)**	**Incongruent accuracy (%)**	**Stroop accuracy effect (%) (congruent - incongruent)**
Healthy controls (n = 20)	954.85 (411.31)	1121.29 (417.31)	166.44 (116.95)	96.88 (5.37)	91.75 (7.35)	5.12 (5.65)
NT1 patients (n = 21)	885.23 (154.36)	1049.67 (182.18)	164.43 (78.22)	95.71 (5.07)	91.55 (7.00)	4.17 (6.39)

Note: Values are means and (Standard deviations); % = percentages; ms = milliseconds. NT1: narcolepsy type 1 patients.

For Food Stroop accuracy, we observed no main effect of Condition (F(1,395) = 0.954, p = 0.335), no main effect of Group (F(1,39) = 0.005, p = 0.947), and no significant Condition * Group effect (F(1,39) = 1.050, p = 0.312).

### Food Stroop fMRI results

**Figure 4:**
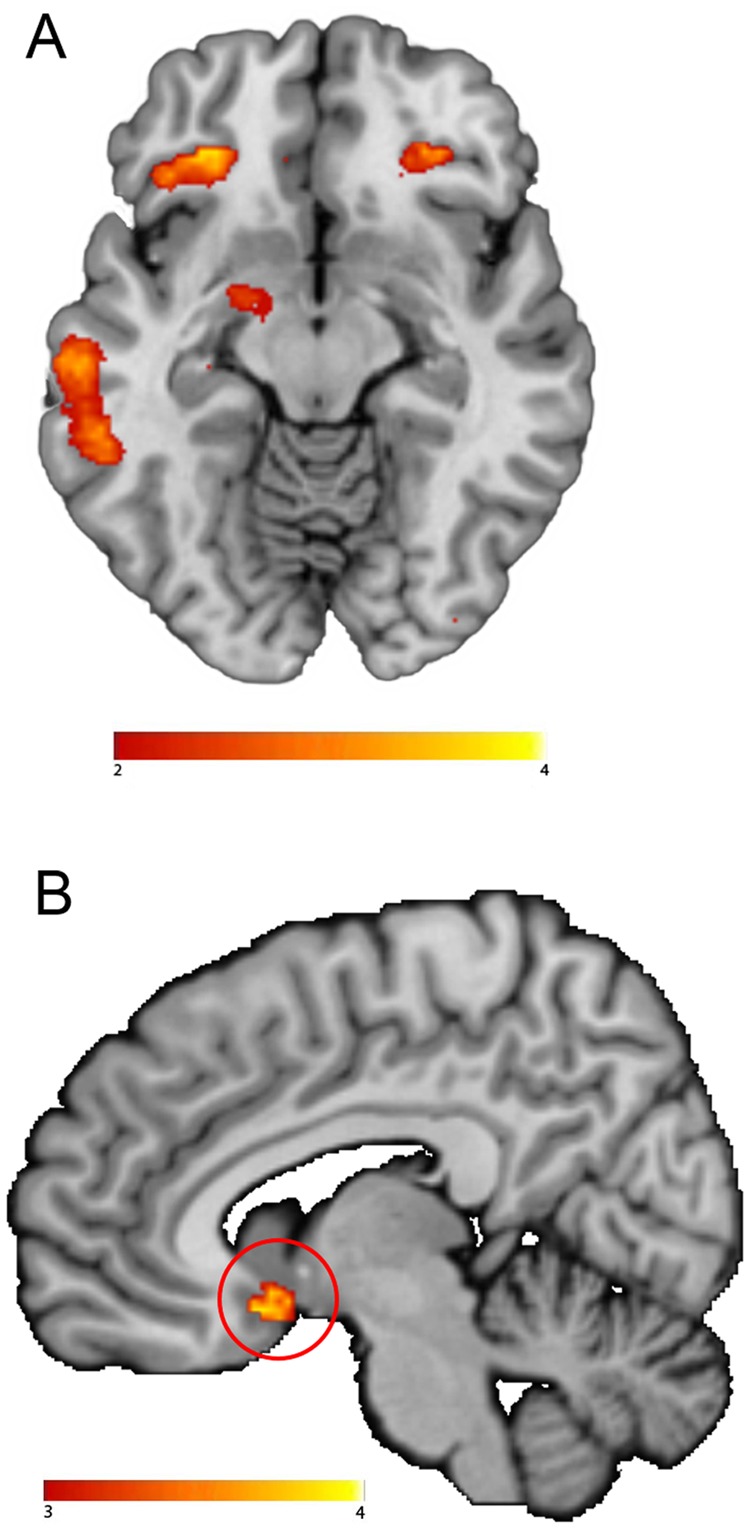
Neural Food Stroop effect. (**A**) Main effect of the contrast of food minus matched neutral words. (**B**) Stronger BOLD response in Narcolepsy type 1 patients versus healthy controls on the food versus neutral words contrast. All statistical parametric maps were overlaid onto a T1-weighted canonical image. Images are shown in neurological convention (left = left). Full brain statistical parametric maps were thresholded at p < 0.001 uncorrected, encircled regions are significant clusters at pFWE < 0.05. Color scale indicates T-scores ranging from 3 (red) to 4 (yellow).

The main task effect of the contrast food words minus neutral words across groups yielded no significant brain responses when applying the pFWE < 0.05 cluster corrected threshold. Using an uncorrected threshold (p < 0.001), we however found similar brain areas as reported in Janssen *et al*.^16^. Namely in the right inferior frontal cortex (Brodmann area 48; x, y, z: 42, 30, 16, t = 4.32, k = 45, p_cluster_uncorrected_ = 0.006), left inferior orbitofrontal cortex (Brodmann area 11; x, y, z: −30, 34,−14, t = 4.10, k = 40, p_cluster_uncorrected_ = 0.009; Fig. 4A) and in the left hippocampus (Brodmann area 20 x, y, z: −30, −20, −18, t = 4.56, k = 21, p_cluster_uncorrected_ = 0.047).

Importantly, on a corrected threshold, NT1 patients displayed increased responses for food versus neutral words in a region of the reward circuitry, i.e. the ventral medial prefrontal cortex (vmPFC/Brodmann area 25; x, y, z: 6, 10,−14, t = 4.45, k = 87, p_cluster-FWE_ = 0.011; Fig. 4B) compared with healthy controls. We did not observe significant correlations with BMI scores nor snack intake, within or across groups. If anything, snack intake correlated positively with a cluster in the vmPFC at p < 0.001 uncorrected, but this did not survive multiple comparison correction (x, y, z: 26, 18, −16, t = 4.18, k = 28, p_cluster-FWE_ = 0.543).

### Functional connectivity with the vmPFC seed during the Food Stroop task

**Figure 5:**
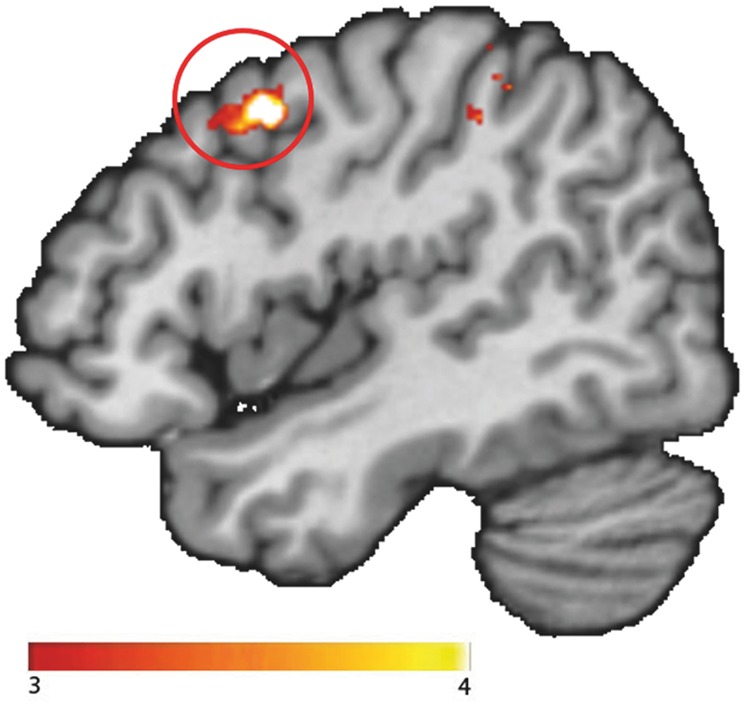
Functional connectivity between the vmPFC seed and the right motor cortex was higher in Narcolepsy type 1 patients. All statistical parametric maps were overlaid onto a T1-weighted canonical image. Images are shown in neurological convention (left = left). Full brain statistical parametric maps were thresholded at p < 0.001 uncorrected, encircled regions are significant clusters at pFWE < 0.05. Color scale indicates T-scores ranging from 3 (red) to 4 (yellow).

We found stronger functional connectivity for NT1 patients relative to healthy controls between the vmPFC seed and the right premotor cortex during the Food Stroop task (food – neutral trials) (Brodmann area 6; x, y, z: 46, 8, 42, t = 4.48, k = 70, p_cluster-FWE_ = 0.032; Fig. 5). This functional connectivity was not related to spontaneous snack intake or BMI.

### Behavioral performance on the Classic Stroop task

To check whether any observed differences in the Food Stroop task or in snack intake could be due to general executive control deficits, we employed the Classic Stroop task. As expected, participants were faster on the congruent trials than on the incongruent trials (main Condition: F(1,39) = 11.691, p = 0.001). We did not see a main effect of Group (F(1,39) = 0.533, p = 0.470), and no significant Condition * Group effect on RTs (F(1,39) = 0.004, p = 0.949) (Table 2). Participants were also more accurate on the congruent versus the incongruent trials (main Condition: F(1,39) = 4.097, p = 0.05). We did not see group differences across trials (main Group: F(1,39) = 0.157, p = 0.694) or as a function of congruency (Condition * Group: F(1,39) = 0.258, p = 0.614) (Table 2).

### Classic Stroop fMRI results

**Figure 6:**
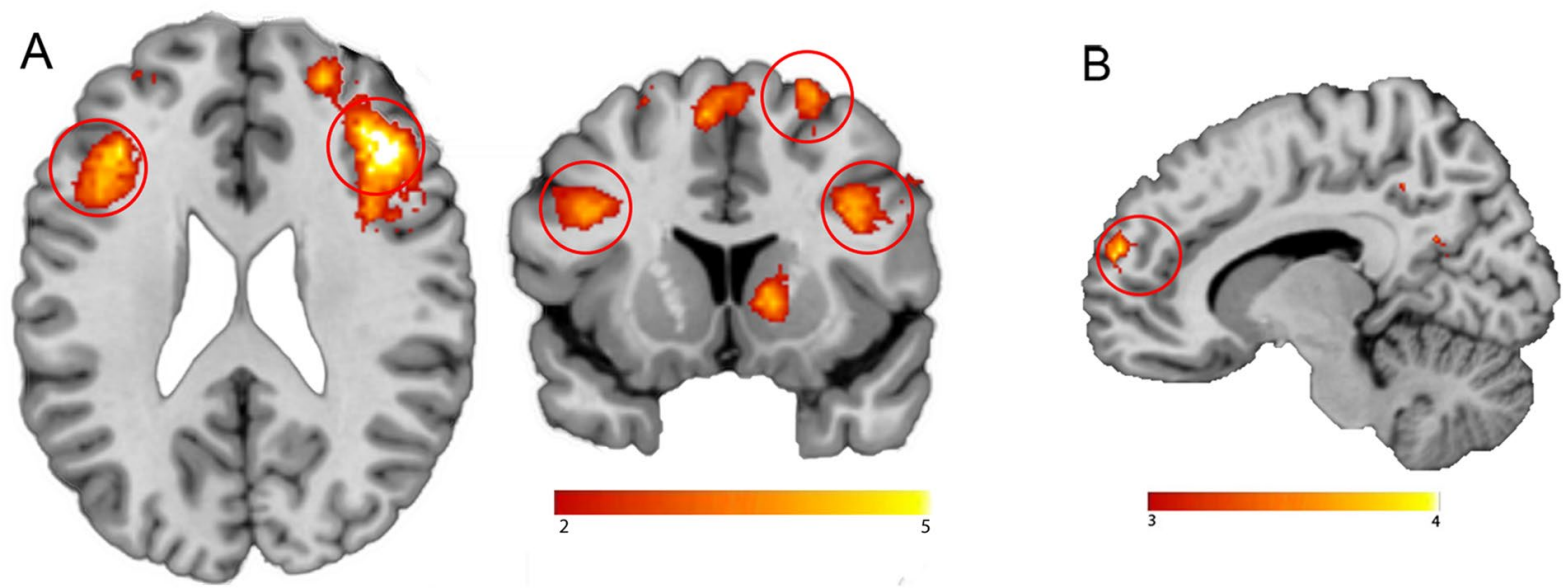
Neural Classic Stroop effect. (**A**) Main effect across groups on the incongruent versus congruent words contrast in the classic Stroop task. Color scale indicates T-scores ranging from 2 (red) to 5 (yellow). (**B**) Stronger BOLD response in healthy controls versus Narcolepsy Type 1 patients on the incongruent versus congruent words contrast. All statistical parametric maps were overlaid onto a T1-weighted canonical image. Images are shown in neurological convention (left = left). Full brain statistical parametric maps were thresholded at p < 0.001 uncorrected (for illustration purposes), encircled regions are significant clusters at pFWE < 0.05. Color scale indicates T-scores ranging from 3 (red) to 4 (yellow).

The main task effect of the contrast incongruent words minus congruent words across groups resulted in significant clusters (Fig. 6A) in the bilateral inferior frontal cortex (right x, y, z: 40, 26, 22, t = 5.89, k = 773, p_cluster-FWE_ < 0.001; left x, y, z: −36, 24, 20, t = 5.02, k = 725, p_cluster-FWE_ < 0.001), supplementary motor cortex (x, y, z: −6, 14, 54, t = 4.87, k = 201, p_cluster-FWE_ < 0.001), right superior frontal cortex (x, y, z: 26, 12, 60, t = 4.82, k = 233, p_cluster-FWE_ < 0.001) and left middle frontal cortex (x, y, z: −26, −10, 52, t = 4.75, k = 85, p_cluster-FWE_ = 0.014). Compared with healthy controls, NT1 patients displayed lower responses for incongruent words minus congruent words in the left dorsal medial prefrontal cortex (dmPFC) (superior frontal gyrus/ Brodmann area 32; x, y, z: −18, 38, 34, t = 5.73, k = 191, p_cluster-FWE_ < 0.001: Fig. 6B).

### Relative contribution of neural Stroop responses in predicting food intake

**Figure 7:**
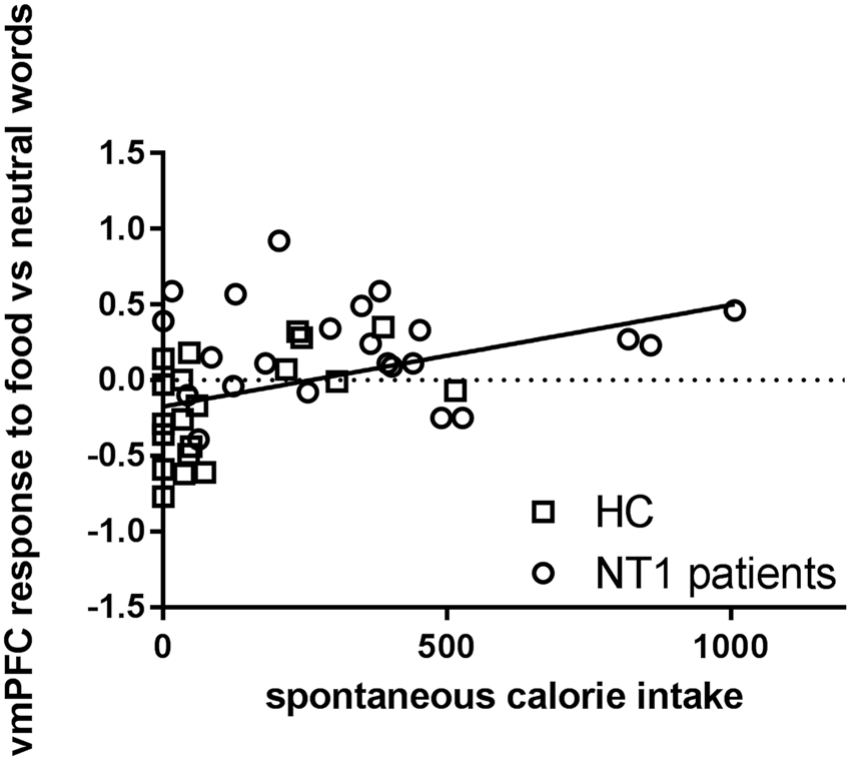
Visual presentation of the relative contribution of vmPFC responses during the Food Stroop task to spontaneous snack intake in the healthy controls and Narcolepsy type 1 patients.

To assess the relative contribution of the responses in the vmPFC responses during the Food Stroop task and in the dmPFC during the Classic Stroop task to spontaneous snack intake, we performed a multiple linear regression analysis across groups to predict spontaneous snack intake based one the beta values extracted from the vmPFC and dmPFC clusters. A trending regression equation was found (F(2,38) = 3.127, p = 0.056), with an R^2^ of 0.148. Only the vmPFC responses elicited on the Food Stroop task were a significant positive predictor of spontaneous snack intake (standardized Beta = 0.330, t = 2.086, p = 0.043; Fig. 7), whereas the dmPFC responses on the Classic Stroop task were not significantly correlated to spontaneous snack intake (standardized Beta = −0.138, t = −0.875, p = 0.388). Thus, food reward-related vmPFC responses on the Food Stroop task, which were increased in NT1 patients versus healthy controls, have a relatively larger contribution to spontaneous snack intake than the executive functioning-related dmPFC cortex responses, which were decreased in narcolepsy patients versus healthy controls. A similar multiple linear regression within NC, did not result in a significant regression equation. Brain responses in the vmPFC and dmPFC did not significantly predict BMI scores.

### Sensitivity analyses including only patients with known orexin levels

For 13 NT1 patients, orexin cerebrospinal fluid levels were known, so orexin deficiency was confirmed. Although analyses with this sub-sample are necessarily underpowered, we performed a sensitivity analysis to show whether our main behavioural and fMRI findings would be similar as in our complete narcolepsy sample.

Behaviorally, similar as in the complete sample, participants did not respond faster to food words than to neutral words (main effect of Condition: F(1,32) = 2.464, p = 0.126). We did not see a main effect of Group: (F(1,32) = 2.809, p = 0.103), and no significant Condition * Group effect on RTs (F(1,32) = 0.465, p = 0.500). For Food Stroop accuracy, we also observed no main effect of Condition (F(1,32) = 0.039, p = 0.844), no main effect of Group (F(1,32) = 0.436, p = 0.514), and no significant Condition * Group effect (F(1,32) = 0.340, p = 0.564).

In the fMRI analyses of the Food Stroop, we again found similar results as in the full sample, albeit weaker and non-significant when correcting at the whole-brain level. NT1 patients compared to controls showed enhanced vmPFC activity (x, y, z, 12, 18, −12, t = 3.87, k = 8, p_cluster-FWE_ = 0.990, p_uncorrected_ = 0.001). No other group differences were found.

Behaviorally on the Classic Stroop, we observed again the same results as with the complete sample; participants were faster on the congruent trials than on the incongruent trials (main Condition: F(1,31) = 110.761, p < 0.001). We did not see a main effect of Group (F(1,31) = 0.712, p = 0.390), and no significant Condition * Group effect on RTs (F(1,31) = 0.096, p = 0.759). Participants were also more accurate on the congruent versus the incongruent trials (main Condition: F(1,31) = 21.269, p < 0.001). We did not see group differences across trials (main Group: F(1,31) = 0.007, p = 0.935) or as a function of congruency (Condition * Group: F(1,31) = 0.288, p = 0.596).

In the fMRI analyses of the Classic Stroop, we again found similar results as in the full sample. Compared with healthy controls, NT1 patients displayed lower responses for incongruent words minus congruent words in the left dorsal medial prefrontal cortex (dmPFC) (superior frontal gyrus/ Brodmann area 32; x, y, z: −18, 38, 34, t = 4.49, k = 26, p_cluster-FWE_ = 0.022). No other group differences were found.

### Control comparisons

We additionally compared NT1 patients with a control group of patients with idiopathic hypersomnia (IH, n = 15, see Supplementary Information Table 1), to verify that our findings in narcolepsy patients were not solely attributable to possible decreased alertness and medication-withdrawal (all patients were at least 1 week off medication).

As in the comparison with healthy controls, NT1 patients consumed significantly more calories than IH patients (mean: 80.85 SD: 126.56; F(1,37) = 12.086, p = 0.001) after the task.

Similar to the comparison between healthy controls and NT1 patients, during the Food Stroop task, there were no differences in behavioural performance (Table 2 in the Supplementary Information) between NT1 patients and IH patients, but the vmPFC region (vmPFC/Brodmann area 25; x, y, z: 8, 14, −12, t = 5.07, k = 149, p_cluster-FWE_ < 0.001) and the left superior temporal lobe (Brodmann area 48; x, y, z: −44, −12, −08, t = 5.36, k = 248, p_cluster-FWE_ < 0.001) were more active in NT1 compared with IH patients. Moreover, we did not observe significant whole brain correlations with BMI scores nor snack intake, within or across NT1 and IH patients. In contrast to the NT1 versus HC comparisons, we found no significant between-group differences in functional connectivity with the vmPFC when comparing narcolepsy patients with IH patients.

During the Classical Stroop task there were no differences in behavioural performance nor in brain responses between NT1 patients and IH patients (Table 2 in the Supplementary Information).

To assess the relative contribution of the responses in the vmPFC responses during the Food Stroop task and in the dmPFC during the Classic Stroop task to spontaneous snack intake, we also performed a multiple regression analysis. A regression model was found (F(2,33) = 2.868, p = 0.072), with R^2^ of 0.156. Only the vmPFC response elicited on the Food Stroop task revealed itself as a trending positive predictor of spontaneous snack intake (standardized Beta = 0.293, t = 1.769, p = 0.087), whereas the dmPFC response on the Classic Stroop task was not significantly correlated to spontaneous snack intake (standardized Beta = −0.244, t = −1.473, p = 0.151). Brain responses in the vmPFC and dmPFC did not significantly predict BMI scores.

A direct comparison between IH patients and controls revealed no behavioral differences on the Food Stroop and no differences in brain responses. On the Classic Stroop there were no behavioral differences, but IH patients displayed a decreased response in the posterior cingulate cortex compared with healthy controls (x, y, z: −4, −42, 20, t = 4.78, k = 80, pcluster-FWE = 0.010).

## Discussion

In this study we aimed to elucidate the role of orexin in neurocognitive mechanisms underlying food attentional bias by investigating NT1 patients, as a orexin-deficient ‘model’. NT1 patients, compared with healthy controls. showed increased activation of the vmPFC when responding to food words relative to neutral words, and displayed higher vmPFC connectivity with the motor cortex when doing so.

The vmPFC is part of the fronto-striatal reward circuitry and is often found to show enhanced activity when people are cued with high caloric food cues (e.g. pictures or words) versus low caloric food cues^9,10,12,32,33^. Indeed, enhanced reactivity to food cues has been shown to predict future weight gain in healthy weight individuals^13,14^. For example, Stice and colleagues^15^ found that elevated vmPFC/orbitofrontal cortex responses to cues signaling impending milkshake receipt predicted future body fat gain over 3-years follow-up in healthy weight adolescents. The current finding of enhanced vmPFC responses in response to rewarding food (versus neutral) words and the enhanced functional connectivity between vmPFC and motor cortex in narcolepsy patients, suggests that narcolepsy patients have enhanced reward-driven invigoration in response to food words which could underlie their weight gain over time. This is further supported by our findings that the vmPFC responses to food (versus neutral) words were a positive and unique predictor (relative to dmPFC responses for incongruent versus congruent words) of spontaneous snack intake after scanning in a marginally significant regression model. However, caution should be warranted in interpreting these latter results, as this was found to be significant across groups but not when only tested within narcolepsy patients. The absence of a significant relation between vmPFC and voluntary snack intake in NT1 patients only might be a power issue or indicate a non-linear relationship between snack intake and vmPFC responses in NT1 (e.g. with vmPFC responses reaching a plateau).

Enhanced responses to food cues have been associated with increased dopamine release in reward-related brain areas^34,35^. Similarly, animal studies have demonstrated that, in response to salient events, orexin projections enhance dopamine firing rates in reward-related areas, including the vmPFC, nucleus accumbens and the dopaminergic ventral tegmental area (VTA)^19,36^. Since NT1 is characterized by orexin deficiency, a *decrease* (via lower VTA activity) instead of an increase in activity of reward-related brain areas might have been expected. Indeed, in a study with monetary reward cues, narcolepsy patients relative to healthy controls, lacked VTA and vmPFC activity when prompted with high versus low incentive monetary cues^37^. Presently, we only observed enhanced vmPFC responses and connectivity for (food) reward-related stimuli and no accompanying diminished responses in other reward regions. How orexin deficiency in narcolepsy patients exactly relates to enhanced vmPFC activity in response to food stimuli requires further study.

On the general executive control task, e.g. Classic Stroop, NT1 patients displayed lower responses in dmPFC for incongruent colored words versus congruent colored words than healthy controls. The dmPFC is part of the executive control network and is sensitive to the degree of response conflict^38–40^. Behaviorally, there were no significant group differences on the Stroop interference effect, which is in line with previous cognitive studies assessing executive functioning in narcolepsy^41,42^. It is less likely that diminished executive control responses in dorsal frontal regions in NT1 lead to overeating, as we currently did not find a relation with snack intake. Instead, general sleepiness in both patient groups might be related to diminished responses in these executive control/attention regions, as shown before during sleep deprivation^43–45^.

One caveat of this study is the absence of expected Food Stroop main effects, in both behavioural and fMRI responses. A previous fMRI study used the same Food Stroop task to measure attentional bias to food words relative to neutral words in healthy controls (n = 76, 85% women, BMI: 19–35)^16^. They reported activation patterns in frontal-parietal areas (including the inferior frontal cortex, inferior orbitofrontal cortex and middle temporal cortex) and slower reaction times (i.e. indicating interference of food words) when healthy controls responded to food relative to neutral words. On a lower statistical threshold (p < 0.001 uncorrected), we indeed find similar brain areas for the food vs neutral contrast as reported in Janssen *et al*.^16^. The decrease in power might be due to the fact that our participants were less weight concerned than the subjects of Janssen *et al*.^16^, who all signed up for an intervention study to change eating habits. Indeed, individuals who are preoccupied with a healthy weight also show increased behavioral food attentional bias^46,47^. Moreover, our study included both patients and healthy volunteers, with the healthy controls showing - if anything - RT interference by the food words (as in Janssen *et al*.^16^), whereas the patients demonstrated - if anything - RT facilitation by food words (see Fig. 2). Although these opposite behavioural effects in patients versus controls did not reach significance, they could have resulted in the absence of main task effects. Importantly, our study was able to pick up enhanced vmPFC responses in narcolepsy patients relative to healthy controls. In addition to healthy controls, we used an extra control group of n = 15 IH patients to discern sleep-disorder related issues (like excessive sleepiness and medication withdrawal) from orexin-deficiency effects. On the Food Stroop task, narcolepsy patients showed enhanced vmPFC responses relative to IH patients, similar to when comparing narcolepsy patients with HC. This suggests that it is unlikely that decreased alertness or medication withdrawal alone would explain our enhanced vmPFC response in narcolepsy versus controls. Similar to narcolepsy patients, IH patients showed normal Stroop behavior but displayed lower responses in the middle cingulate cortex relative to healthy controls, which is also part of the executive control network. Hence, general sleepiness in both patient groups might be related to diminished responses in these executive control/attention regions, as shown previously during sleep deprivation^43–45^. However, the additional IH control group was small and not well matched to the NC patients, so this comparison should be interpreted with caution. We tried to minimize the confounding factor of medication effects by having people abstain from medication use 7 days before testing. Nevertheless, different treatments may have differentially affected neuronal mechanisms important for obesity and appetite^48^ and drug withdrawal may alter this complex relation. Larger sample sizes and longitudinal studies are needed to systematically dissect the specific medication effects on obesity and appetite- associated neuronal processes. Due to practical reasons, such as participant- and MRI scan availability, not all participants were tested at the same time. As circadian rhythms influence appetite, we did balance the timing and fasting between the groups, but future studies should further minimize circadian differences between groups.

Finally, although all patients had a definite NT1 diagnosis, including clear-cut cataplexy as assessed by an narcolepsy expert, we did not have orexin levels of each narcolepsy patient in our complete narcolepsy sample. Therefore, we included additional sensitivity analyses only including the 13 NT1 patients with available orexin levels to see whether our findings would still remain. These analyses showed that, although this subsample was understandably underpowered, the results are comparable to the full sample. This further validates the main results and strengthens our notion that the NT1 sample is suitable as a ‘model’ to study effects of orexin deficiency on the group level.

Our study is the first to demonstrate neurocognitive mechanisms of food cues processing in NT-1 patients, indicating abnormal food-related motivational brain responses in a state of orexin deficiency, which may contribute to overeating in this disorder.

## Electronic supplementary material

Supplementary Information

## Data Availability

The datasets generated during and/or analysed during the current study are available from the corresponding author on reasonable request.
