# Publisher Correction: Enhanced food-related responses in the ventral medial prefrontal cortex in narcolepsy type 1

**DOI:** 10.1038/s41598-019-44073-x

**Published:** 2019-06-14

**Authors:** Ruth Janke van Holst, Lieneke K. Janssen, Petra van Mierlo, Gert Jan Lammers, Roshan Cools, Sebastiaan Overeem, Esther Aarts

**Affiliations:** 1 Department of Neurology, Radboud university medical center, Nijmegen, The Netherlands; 2 Donders Institute for Brain, Cognition and Behaviour, Radboud University, Nijmegen, The Netherlands; 3 Sleep Medicine Center Kempenhaeghe, Heeze, The Netherlands; 4 Sleep-Wake Center SEIN, Heemstede, The Netherlands; 5 Department of Neurology, Leiden University Medical Center, Leiden, The Netherlands; 6 Department of Psychiatry, Radboud university medical center, Nijmegen, The Netherlands; 7 Eindhoven University of Technology, Eindhoven, The Netherlands; 8 Department of Psychiatry, Amsterdam UMC, University of Amsterdam, Amsterdam, The Netherlands

Correction to: *Scientific Reports* 10.1038/s41598-018-34647-6, published online 06 November 2018

In the original version of this Article, the author Gert Jan Lammers was incorrectly indexed. This error has now been corrected.

