# Supplementary material for: Enhanced food-related responses in the ventral medial prefrontal cortex in narcolepsy type 1
Source: Sci Rep. 2018 Nov 6;8:16391. doi: 10.1038/s41598-018-34647-6 (PMC6219562; doi:10.1038/s41598-018-34647-6)
Supplement: Supplementary file 1 — Supplementary Information [file 41598_2018_34647_MOESM1_ESM.pdf]

Title Page: Supplementary information

Title: Enhanced food-related responses in the ventral medial prefrontal cortex in narcolepsy type 1.

Sub-title: Food-related responses in narcolepsy

Authors: Ruth Janke van Holst*[1,2,8], PhD., Lieneke K. Janssen[2], PhD., Petra van Mierlo[3], Msc., Gert Jan Lammers[4,5], MD, PhD., Roshan Cools[2,6], PhD., Sebastiaan Overeem[3,7^],MD, PhD., Esther Aarts[2^],PhD.

^ These authors contributed equally to this work

Affiliations:

[1]Dept. of Neurology, Radboud university medical center, Nijmegen, the Netherlands

[2]Donders Institute for Brain, Cognition and Behaviour, Nijmegen, the Netherlands

[3]Sleep Medicine Center Kempenhaeghe, Heeze, the Netherlands

[4]Sleep-Wake Center SEIN, Heemstede, the Netherlands

[5]Dept. of Neurology, Leiden University Medical Center, Leiden, the Netherlands

[6]Dept. of Psychiatry, Radboud university medical center, Nijmegen, the Netherlands

[7]Eindhoven University of Technology, Eindhoven, the Netherlands

[8]Dept. of Psychiatry, Amsterdam UMC, University of Amsterdam, Amsterdam, Netherlands

Corresponding author: Ruth J. van Holst, Amsterdam UMC, University of Amsterdam, Department of Psychiatry, room: PA3-226, P.O. box 22660 1100 DD Amsterdam The Netherlands, Telephone number: +31 (0)20 8913760, email address: mail@ruthvanholst.nl

Supplementary Information

Table1 Demographic and clinical characteristics of the idiopathic hypersomnia patients

	**IH patients (n=15)**	**STATS** NT1 vs IH:
**Male/ Female**	7/8	p=0.740
**Age**	36.20 (12.89)	p=0.494
**Total score Digit Span**	15.47 (2.82)	p=0.751
**Education levels:**	2.86 (0.64)	p=0.790
**BMI**	24.22 (4.72)	p=0.089
**Disease duration**	6.40 (8.13)	p=0.520
**ESS**	15.66 (3.48)	p=0.751
**PSQI**	7.54 (3.01)	p=0.863
**Medication used:**		
**- Stimulants #**	11	p=0.294
**-Anti-depressants #**	0	p=0.413
**-Sodium oxybate #**	1	p=0.531

**-Stimulants plus sodium oxybate #**	0	p=0.241
**-No medication#**	1	p=0.339

Note. Variables are reported as mean and (standard deviations). Disease duration is reported in mean years. Education levels were categorized as 1= Lower Vocational Education, 2= Intermediate Vocational, 3= Higher Vocational, 4= University; BMI= Body Mass Index; ESS= Epworth Sleepiness Scale; PSQI= Pittsburgh Sleep Quality Index; #= number of participants; *=Significant at < 0.05; Group differences on age, gender, education level and medication use were tested with an Chi-square test. Other tests were F-tests. NT1: narcolepsy type 1 patients; IH: idiopathic hypersomnia patients.

Table 2. Behavioral results from the Food Stroop task and classic Stroop task for the idiopathic hypersomnia patients

**Food Stroop task**	**Food RTs (ms)**	**Neutral RTs (ms)**	**Food Stroop RT effect (ms) (food – neutral)**	**Food accuracy (%)**	**Neutral accuracy (%)**	**Food Stroop accuracy effect (%) (food-neutral)**
**IH patients (n=15)**	833.93 (407.34)	852.96 (425.67)	-19.03 (50.08)	97.36 (2.85)	98.27 (2.37)	-0.33 (3.25)
**Classic Stroop Task**	**Congruent RTs (ms)**	**Incongruent RTs (ms)**	**Stroop RT effect (ms) (incongruent – congruent)**	**Congruent accuracy (%)**	**Incongruent accuracy (%)**	**Stroop accuracy effect (%) (congruent - incongruent)**

**IH patients (n=13)**	869.96 (415.98)	992.05 (383.54)	122.08 (88.08)	98.08 (3.56)	94.23 (4.26)	3.85 (3.33)

Note: Values are means and (Standard deviations); %=percentages; ms= milliseconds. IH: idiopathic hypersomnia patients
